# Supplementary material for: First-in-Human Dose-Escalation Study of the Novel Oral Depsipeptide Class I-Targeting HDAC Inhibitor Bocodepsin (OKI-179) in Patients with Advanced Solid Tumors
Source: Cancers (Basel). 2023 Dec 23;16(1):91. doi: 10.3390/cancers16010091 (PMC10778198; doi:10.3390/cancers16010091)
Supplement: Supplementary file 1 [file cancers-16-00091-s001.zip › cancers-2752789-supplementary.pdf]

Table S1. Treatment-Emergent Adverse Events by Grade in ≥ 15% of Patients Treated With Continuous Dosing of OKI-179

System Organ Class Preferred Term, *N* (%)	200 mg (*N*=6)				300 mg (*N*=2)				All Continuous Patients (*N*=8)				
	**G1**	**G2**	**G3**	**G4**	**G1**	**G2**	**G3**	**G4**	**G1**	**G2**	**G3**	**G4**	**All grades**
Constipation	2 (33)	3 (50)	0	0	1 (50)	0	0	0	3 (38)	3 (38)	0	0	6 (75)
Nausea	2 (33)	2 (33)	0	0	2 (100)	0	0	0	4 (50)	2 (25)	0	0	6 (75)
Fatigue	1 (17)	2 (33)	0	0	0	0	1 (50)	0	1 (13)	2 (25)	1 (13)	0	4 (50)
Platelet count decreased	1 (17)	1 (17)	0	0	0	0	0	2 (100)	1 (13)	1 (13)	0	2 (25)	4 (50)
Diarrhea	2 (33)	0	0	0	1 (50)	0	0	0	3 (38)	0	0	0	3 (38)
Headache	2 (33)	0	0	0	1 (50)	0	0	0	3 (38)	0	0	0	3 (38)
Decreased appetite	1 (17)	0	0	0	0	0	1 (50)	0	1 (13)	0	1 (13)	0	2 (25)
Anemia	0	1 (17)	0	0	0	1 (50)	0	0	0	2 (25)	0	0	2 (25)
Abdominal pain	1 (17)	1 (17)	0	0	0	0	0	0	1 (13)	1 (13)	0	0	2 (25)
Dyspepsia	1 (17)	1 (17)	0	0	0	0	0	0	1 (13)	1 (13)	0	0	2 (25)
ECG QT prolonged	1 (17)	1 (17)	0	0	0	0	0	0	1 (13)	1 (13)	0	0	2 (25)
Pain	1 (17)	1 (17)	0	0	0	0	0	0	1 (13)	1 (13)	0	0	2 (25)

Graded using National Cancer Institute Common Terminology Criteria for Adverse Events (CTCAE), version 5. Abbreviations: Gr, Grade; *N*, Number. There were no Grade 5 TEAEs in patients treated with continuous dosing.

Table S2. Treatment-Emergent Adverse Events by Grade in ≥ 15% of Patients Treated With Intermittent Dosing of OKI-179 of 5 Days on / 2 Days Off (5:2)

**Adverse Event** *N* **(%)**	**300 mg (*N*=6)**			
	**G1**	**G2**	**G3**	**All grades**
Nausea	4 (67)	0	1 (17)	5 (83)
Fatigue	1 (17)	3 (50)	0	4 (67)
Platelet count decreased	4 (67)	0	0	4 (67)
Vomiting	2 (33)	1 (17)	0	3 (50)
Decreased appetite	0	1 (17)	1 (17)	2 (33)
Anemia	1 (17)	0	1 (17)	2 (33)
Back pain	1 (17)	0	1 (17)	2 (33)
ECG QT prolonged	2 (33)	0	0	2 (33)
Bile duct stenosis	0	0	1 (17)	1 (17)
Enterococcal bacteremia	0	0	1 (17)	1 (17)
Pneumothorax	0	0	1 (17)	1 (17)
Abdominal pain	0	1 (17)	0	1 (17)
Dysphagia	0	1 (17)	0	1 (17)
Esophagoscopy	0	1 (17)	0	1 (17)
Oropharyngeal pain	0	1 (17)	0	1 (17)
Pain in extremity	0	1 (17)	0	1 (17)
Pneumonitis	0	1 (17)	0	1 (17)
Aspartate aminotransferase increased	1 (17)	0	0	1 (17)
Blood alkaline phosphatase increased	1 (17)	0	0	1 (17)
Dehydration	1 (17)	0	0	1 (17)
Eye pain	1 (17)	0	0	1 (17)
Gastritis	1 (17)	0	0	1 (17)
Hypomagnesaemia	1 (17)	0	0	1 (17)
Myalgia	1 (17)	0	0	1 (17)
Neutrophil count decreased	1 (17)	0	0	1 (17)
Esophagitis	1 (17)	0	0	1 (17)
Paresthesia	1 (17)	0	0	1 (17)

Graded using National Cancer Institute Common Terminology Criteria for Adverse Events (CTCAE), version 5. Abbreviations: Gr, Grade; *N*, Number. There were no Grade 4 or Grade 5 TEAEs in patients treated with the 5:2 schedule.

Table S3. Summary of Key PK Parameters for the 300-mg 5:2 Regimen Cohort

Analyte	Cycle 1 Visit	C_{max} (ng/mL)	T_{max} (hr)	AUC_{last} (hr*ng/mL)	$t_{1/2}$ (hr)	MR_{AUC}
OKI-179	Day 1	142 (31.2%)	0.875 (0.517 - 2.00)	253 (101%)	1.75 (0.672 - 2.83)	NA
	Day 15	215 (77.1%)	0.934 (0.550 - 1.93)	374 (126%)	1.74 (1.09 - 2.40)	NA
OKI-006 (Free)	Day 1	1770 (37.3%)	0.692 (0.500 - 1.05)	2790 (61.6%)	NA	13.3 (59.7%)
	Day 15	2220 (38.9%)	0.934 (0.500 - 1.93)	3180 (60.3%)	3.43 (0.994 - 6.64)	10.3 (52.4%)
OKI-006 (Total)	Day 1	1990 (36.2%)	0.942 (0.533 - 1.90)	6040 (60.6%)	6.70 (6.14 - 7.07)	28.8 (65.2%)
	Day 15	2420 (33.4%)	0.934 (0.500 - 1.93)	6420 (44.2%)	6.53 (6.30 - 9.68)	20.7 (76.0%)

Values are geometric mean (%CV) for C_{max}, AUC_{last} and MR_{AUC} for N=6 patients in the 300-mg 5:2 regimen cohort. Values are median (min-max) for T_{max} and terminal $t_{1/2}$. NA=not applicable. MR_{AUC} is the OKI-006 to OKI-179 metabolite ratio based on AUC_{last}.

Table S4. Mean Acetylation Fold Change from Baseline at 2 Hours Post-dose

	H3K9			H3K27			Total Lysine		
**Dose (mg)**	**CD4**	**CD8**	**T-cell**	**CD4**	**CD8**	**T-cell**	**CD4**	**CD8**	**T-cell**
30	1.4 (NA)	1.5 (NA)	1.4 (NA)	4.8 (NA)	5.2 (NA)	7.4 (NA)	1.6 (NA)	1.2 (NA)	1.6 (NA)
60	1.6 (0.6)	1.8 (0.6)	2.0 (0.8)	6.0 (3.4)	3.7 (1.9)	4.3 (4.3)	1.4 (NA)	1.2 (NA)	1.3 (NA)
90	1.4 (NA)	2.4 (NA)	2.2 (NA)	2.1 (NA)	4.1 (NA)	4.2 (NA)	0.8 (NA)	1.8 (NA)	1.6 (NA)
120	2.5 (NA)	2.3 (NA)	2.5 (NA)	10.2 (NA)	10.1 (NA)	9.3 (NA)	3.4 (NA)	4.0 (NA)	3.4 (NA)
180	3.3 (NA)	2.6 (NA)	3.1 (NA)	8.8 (NA)	6.2 (NA)	8.0 (NA)	3.0 (NA)	2.2 (NA)	2.7 (NA)
200	2.2 (1.7)	2.3 (1.8)	2.4 (1.8)	3.7 (2.5)	4.0 (2.0)	4.3 (2.5)	2.1 (1.3)	1.9 (1.3)	2.3 (1.4)
210	3.6 (NA)	3.7 (NA)	3.8 (NA)	10.6 (NA)	10.6 (NA)	10.9 (NA)	4.0 (NA)	3.5 (NA)	3.7 (NA)
270	2.5 (0.7)	2.4 (0.5)	2.5 (0.6)	8.0 (2.9)	6.6 (1.8)	6.8 (2.3)	2.9 (0.7)	2.6 (0.4)	2.8 (0.5)
300	1.6 (1.2)	1.4 (0.7)	1.5 (0.8)	6.2 (4.3)	5.4 (2.9)	5.9 (3.3)	2.7 (2.2)	2.5 (1.7)	2.7 (1.6)
350	3.2 (0.1)	1.0 (0.1)	2.9 (0.1)	10.8 (0.9)	1.8 (0.3)	9.6 (1.0)	4.0 (0.1)	2.0 (NA)	3.9 (0.1)
450	4.2 (2.9)	4.5 (2.2)	3.3 (1.9)	9.7 (5.2)	8.8 (4.1)	8.5 (3.5)	3.2 (2.3)	2.6 (1.5)	3.0 (2.1)

Values are mean with standard deviation. NA=not applicable due to limited number of values.
